# Characterization of brain transduction capability of a BBB-penetrant AAV vector in mice, rats and macaques reveals differences in expression profiles

**DOI:** 10.1038/s41434-024-00466-w

**Published:** 2024-07-22

**Authors:** María Bunuales, Angeles Garduno, Miguel Chillon, Assumpció Bosch, Manuela Gonzalez-Aparicio, Maria Espelosin, Marta Garcia-Gomara, Alberto J. Rico, Ana Garcia-Osta, Mar Cuadrado-Tejedor, Jose L. Lanciego, Ruben Hernandez-Alcoceba

**Affiliations:** 1 Division of DNA and RNA medicine, CIMA. University of Navarra, Pamplona, Spain; 2 Institut de Neurociències, Biochemistry and Molecular Biology Department, Universitat Autònoma de Barcelona; and Vall d’Hebron Research Institute, Barcelona, Spain; 3 Institució Catalana de Recerca i Estudis Avançats (ICREA), Barcelona, Spain; 4 Pathology, Anatomy and Physiology Department, School of Medicine, University of Navarra, IdiSNA, 31008 Pamplona, Spain; 5 Centro de Investigación Biomédica en Red de Enfermedades Neurodegenerativas (CiberNed), Madrid, Spain

**Keywords:** Genetic vectors, Genetic transduction

## Abstract

Different screening methods are being developed to generate adeno-associated viral vectors (AAV) with the ability to bypass the blood-brain barrier (BBB) upon intravenous administration. Recently, the AAV9P31 stood out as the most efficient version among a library of peptide-displaying capsids selected in C57BL/6 mice using RNA-driven biopanning. In this work we have characterized in detail its biodistribution in different mouse strains (C57BL/6 and Balb/c), as well as in Sprague Dawley rats and non-human primates (*Macaca fascicularis*). Using GFP and NanoLuc reporter genes, we confirmed homogeneous infection and transgene expression across the CNS of mice injected intravenously with AAV9P31. A more restricted pattern was observed upon either intracerebroventricular or intraparenchymal injection. Following intravenous delivery, region- and cell-specific differential patterns of transduction were observed in the mouse brain, including a preferential transduction of astrocytes and neurons in the cerebral cortex and striatum, whereas neurons were the only transduced cell type in subcortical locations across the hippocampus, thalamus, hypothalamus, mesencephalon, brainstem and cerebellum. Furthermore, transduced microglial cells were never found in any CNS location. Peripheral organs transduced upon intravenous administration included lung, liver, peritoneum, heart and skeletal muscle. However, a comparable performance of AAV9P31 to bypass the BBB in rats and macaques was not observed, although a more limited neuronal transduction was found in the brainstem of rats upon intravenous delivery. Finally, intracerebroventricular delivery in macaques resulted in neuronal transduction in cortical, subcortical structures and cerebellum following a patchy pattern. In conclusion, the widespread CNS transduction obtained in mice upon intravenous delivery of AAV9P31 represents a powerful tool for modeling a wide variety of neurological disorders as well as an appealing choice for the evaluation of gene therapy-based therapeutics.

## Introduction

The development of vectors with the ability to transfer genetic material to the Central Nervous System (CNS) upon systemic administration is a long-awaited need in the fields of neuroscience and biomedicine [1]. Homogeneous and efficient knock-down or over-expression of genes in animal models at different developmental stages would speed up basic and translational research, reducing the need of costly transgenic animals. More importantly, such tools could unravel the potential of gene therapy for human diseases affecting the CNS. Although the brain is a well vascularized organ, the blood brain barrier (BBB) does not allow the traffic of conventional vectors to the interstitial space, where they can establish the required contact with the surface of neurons and other cell populations [2, 3]. Administration into the cerebrospinal fluid (CSF) space can bypass this obstacle [4, 5], but it is also challenging due to the ependymal barrier and the CSF patterns of circulation [6, 7]. Finally, direct intraparenchymal injection is the most invasive route of administration. Although very efficient, the spread of transduction from the injection site depends on the characteristics of the vector and the different brain structures [8]. For instance, large particles such as adenoviral vectors (90 nm in diameter) show better diffusion in basal ganglia than cerebral cortex [9]. In addition, local or regional routes of administration often result in a gradient of gene transfer efficacy, with cells receiving an excess of vector copies in areas close to the injection and others remaining poorly transduced [4, 10].

Adeno-associated viral vectors (AAVs) are currently the gold standard vectors for in vivo gene transfer in the CNS [11]. Despite the relatively small size of their particles (25 nm), intraparenchymal or intra-CSF injection of these vectors cannot penetrate deep enough into the brain parenchyma to reach the entire CNS in large animal models or in patients [12, 13]. This limitation hampers the therapeutic effect in relevant indications, such as genetic encephalopathies [14]. To solve this problem, new strategies have been developed for the generation of capsid libraries and their screening in different models (reviewed in [15]). The versatility of the AAV capsids allows great diversity, obtained by DNA shuffling, mutagenesis or peptide display. This latter approach is becoming very efficient, following the identification of permissive and exposed domains in the capsid proteins [16]. Not surprisingly, capsid variants identified by in vivo screening in a particular animal model do not necessarily show similar CNS tropism in other species or strains. This is the case for the widely used AAV-PHP.B [17] and AAV-PHP.eB [18] variants, which achieved stronger BBB penetration compared with the parental AAV9 in certain mouse strains such as C57BL/6 J (the screening host), FVB/NCrl and DBA/2 J, but not in BALB/c [19]. This is due to the interaction of the displaying peptide with the GPI-anchored Ly6a protein, which is highly expressed in the brain microvasculature only in the permissive mouse strains, and not in other mammals including non-human primates (NHP) [20].

- Using the TRACER platform (Tropism Redirection of AAV by Cell-type-specific Expression of RNA), a vector with unprecedented efficacy for brain transduction was identified in C57BL/6 J mice [21]. The AAV9P31 outperformed the parental AAV9 and previously described variants such as AAV-PHP.eB, showing strong and homogeneous transduction of mouse brain following intravenous (i.v.) administration. The modification incorporated in this capsid consists of an insertion of the heptapeptide WPTSYDA between residues 588 and 589 of the hypervariable surface loop VIII. A recent study has identified carbonic anhydrase IV (CA-IV) as the receptor mediating BBB transcytosis of AAV9P31 [22]. Interestingly, this protein is expressed in the surface of CNS endothelial cells in all vertebrates, including NHPs and humans. However, in vitro experiments performed in HEK293T cells expressing CA-IV receptors from different species showed that the rhesus macaque and human versions were unable to enhance AAV9P31 infectivity, in contrast with the mouse CA-IV [22]. Although this result does not diminish the interest of AAV9P31 as a research tool for mouse brain transduction, it casts doubts on its relevance as a vector for clinical use. In order to clarify this issue, we have directly tested the ability of AAV9P31 to transduce the brain of NHPs following i.v., i.c.v. or intraparenchymal administrations. Intense transgene expression only took place upon local injection of the vector, and to a lesser extent upon i.c.v. administration. The inability to cross the BBB was also observed in Sprague Dawley rats. In contrast, i.v. administration in C57BL/6 J or Balb/c mice achieved wider CNS transduction than i.c.v. injection. We provide here a detailed characterization of vector tropism and transgene expression in CNS and peripheral organs in these mouse strains, as well as identification of different brain populations transduced by the vector. This information can guide the use of the AAV9P31 capsid in basic and preclinical research.

## Materials and methods

### Vector production

AAV vectors were produced, purified and manipulated in the biosafety level 2 facilities at the Universitat Autònoma Barcelona. Briefly, AAV vectors were generated using the triple transfection system in HEK293 cells. After 48 h, AAV vectors were harvested, treated with benzonase (71,206–3, Merck, Kenilworth, NJ, USA), and purified in an iodixanol gradient followed by Amicon 100 KDa to substitute iodixanol to PBS-1X + 5% sucrose + 0,001% pluronics, Titration in vg/mL was done using the Picogreen system [23].

### Administration of vectors in vivo

#### Rodents

Intravenous administration was performed by retro-orbital injection of 2 × 10^13^ vg/kg AAV9P31-CMV-GFP or AAV9P31-CMV-NL in isoflurane-anesthetized 7 weeks-old mice and rats, dissolved in a volume of 100 and 400 µl saline solution, respectively. For stereotactic injection, mice were anesthetized with an intraperitoneal mixture of ketamine and xylazine (80 and 10 mg/kg, respectively) and they were treated with 0.1 mg/kg buprenorphine subcutaneously for analgesia. After placing in a stereotactic frame, the scalp was shaved and disinfected before performing a longitudinal incision along the midline to expose the skull, which was cleaned with iodine and hydrogen peroxide to remove the periosteum and prevent infection. For i.c.v. administration, 2 × 10^11^ vg of the AAV9P31-CMV-GFP vector were injected in the left lateral ventricle in a total volume of 5 µl using a Hamilton syringe (Hamilton Co) following a 0.5 µl/min infusion rate. Coordinates were defined using the bregma point as reference: antero-posterior −0.2 mm; half-side +1.0 mm; dorso-ventral −3.2 mm. For intraparenchymal injection (left striatum) 6 × 10^10^ vg of the AAV9P31-CMV-GFP vector in a total volume of 0.5 µl was injected following a 0.2 µl/min infusion rate. In this case, coordinates were: antero-posterior 0.51 mm; lateral +2 mm; dorso-ventral −3.3 mm. Following injections, the needle was left in place for 2 min before withdrawal, to avoid vector leakage. After surgery, animals were kept under constant monitoring with *ad libitum* access to food and water. Note that in this experiment, control mice were sham-injected.

#### Non-human primates

Two macaques (*Macaca fasciularis*) were used for this study (40 months-old female, 3.2 kg, and 42 months-old one male, 3.8 kg). Intravenous administration of the AAV9P31-CMV-GFP vector (2 × 10^13^ vg/kg in 3.5 ml) was performed via the saphenous vein. For stereotactic injections, surgical anesthesia was induced by intramuscular injection of ketamine (5 mg/kg) and midazolam (5 mg/kg). Local anesthesia was implemented just before surgery with a 10% solution of lidocaine. Analgesia was achieved with a single intramuscular injection of flunixin meglumine (Finadyne, 5 mg/kg) delivered at the end of the surgical procedure and repeated 24 and 48 h post-surgery. A similar schedule was conducted for antibiotic delivery (ampicillin, 0.5 mL/day). Stereotactic coordinates were taken from the atlas of Lanciego and Vázquez [24]. During surgery, target selection was assisted by ventriculography. The AAV9P31-CMV-tdT vector (2 × 10^13^ vg in 1 ml/ventricle) was injected bilaterally in the lateral ventricles with an injection rate of 0.2 ml/min. Stereotaxic coordinates were 2 mm rostral to the anterior commissure (ac), 1.3 mm lateral to midline and 5 mm dorsal to the bi-commissural plane. The AAV9P31-CMV-NL vector was injected in the left putamen nucleus in pulses of 1 μl/min for a total volume of 5 μl of the viral suspension. Stereotaxic coordinates were 0 mm from ac, 11.5 mm lateral to midline and 1 mm dorsal to the bicommissural plane. Once AAV delivery was completed, the needle was left in place for 10 min before withdrawal to minimize reflux of the AAV suspension through the injection tract. After surgery, NHPs were kept under constant monitoring in single cages with *ad libitum* access to food and water until full recovery, then returning to the animal vivarium.

#### Quantitative PCR

Mice were perfused with saline solution before organ extraction. Different CNS structures were dissected and tissues were frozen in liquid Nitrogen and kept at –80°C until processing. DNA was extracted from tissues using the Maxwell RSC Tissue DNA Kit (Promega, Madison, WI, USA), as recommended by the manufacturer. DNA was quantified in a Nanodrop apparatus (Thermo Fisher Scientific, Waltham, MA, USA), and 40 ng of each sample were added in the real-time PCR reaction using a iQTM SYBR^®^ Green Supermix reagent (Bio-Rad, Hercules, CA, USA) in CFX96 Touch^TM^ Real-Time PCR Detection System (Bio-Rad). Standard curves were prepared using AAV genomes. Primers were purchased from Sigma-Merck (St Louis, MI, USA) and were directed to GFP or NanoLuc sequences (GFP forward 5ʹ GAGCGCACCATCTTCTTCAAG 3ʹ, reverse 5ʹ TGTCGCCCTCGAACTTCAC 3ʹ. NanoLuc forward 5ʹ ACAGTCTGCAGGTCTTCACACTCGAAGATTTCG 3ʹ, reverse 5ʹ ATGATGACATGGATGTCGATC 3ʹ).

#### Bioluminescence imaging (BLI)

Mice were anesthetized with an intraperitoneal injection of ketamine/xylazine mixture (80:10 mg/kg), and the NanoGlo in vivo substrate fluorofurimazine (Promega) was injected intraperitoneally (40 µl diluted 1:2 in PBS). Light emission was detected 10 min later using a PhotonImager^TM^ Optima apparatus (BioSpace). Data were analyzed using the M3Vision software (BioSpace). Regions of interest (ROIs) were established in the head of mice during a dorsal exposure, whereas light emission from the abdominal and thoracic regions was acquired during a separate exposure with the animals facing the camera.

#### Analysis of luciferase activity in tissue extracts

Samples were obtained as described for qPCR analysis. Tissues were homogenized in 200 µl of PBS supplemented with Complete protease inhibitor cocktail (Roche, Basel, Switzerland) and centrifuged at 11.000 g for 5 min. Ten µl of the supernatants were mixed with 70 µl of PBS, and NanoLuc activity was measured using the Nano-Glo Luciferase Assay System (Promega). Light emission was quantified in a GloMax discover 96 microwell plate reader (Promega).

#### Necropsies and tissue processing

In all animal subjects (mice, rats and NHPs), anesthesia was firstly induced with an intramuscular injection of ketamine (10 mg/Kg), followed by a terminal overdose of sodium pentobarbital (200 mg/Kg) and perfused transcardially with an infusion pump. All animals were sacrificed 4 weeks post-AAV deliveries. The perfusates consisted of a saline Ringer solution followed by a fixative solution made of 4% paraformaldehyde and 0.1% glutaraldehyde in 0.1 M phosphate buffer (PB) pH 7.4. Perfusion was continued with a cryoprotectant solution containing 10% glycerin and 1% dimethylsulfoxide (DMSO) in 0.1 M PB pH 7.4. Once perfusion was completed, the skull was opened and the brains removed and stored for 48 h in a cryoprotectant solution made of 10% glycerin and 2% DMSO in 0.1 M PB pH 7.4. Next, frozen coronal sections (40 μm-thick) were obtained on a sliding microtome and collected in 0.1 M PB pH 7.4 as 10 series of adjacent sections. These series were used for (1) immunoperoxidase detection of GFP, (2) immunoperoxidase with anti-mCherry antibody to detect tdTomato (tdT), (3) immunoperoxidase detection of NL (HA-tagged), (4) immunofluorescent detection of GFP, GFAP and NeuN, (4) immunofluorescent detection of GFP, Iba-1 and NeuN. The remaining 6 series of sections were stored at −80 °C cryopreserved in 20% glycerin and 2% DMSO until further use, if needed. Immunoperoxidase-stained sections were digitalized on an Aperio CS2 slide scanner (Leica, Wetzlar, Germany) at a magnification of 20x, whereas confocal microscopy was used for analysis of immunofluorescent-stained sections. A complete list of primary and bridge antisera (either biotinylated or dye-tagged) together with incubation concentrations, incubation times and commercial sources is provided below in Table 1. Tissue processing and data analysis was carried out by blinded team members.

**Table 1:** List of antibodies used in the study.

Antibody	Host	Dilution	Incubation time	Source	Cat. No.
GFP	Chicken	1:1000	Overnight	Abcam	AB13970
mCherry (tdTomato)	Rabbit	1:1000	Overnight	Abcam	AB167453
HA-tag (C29F4)	Rabbit	1:1600	Overnight	Cell Signaling	3724 S
GFAP	Rabbit	1:400	Overnight	Dako	Z0334
Iba-1	Rabbit	1:500	Overnight	Fujifilm Wako	019-19741
NeuN	Mouse	1:100	Overnight	Millipore	MAB377
Anti-Chicken (Biotin-SP)	Goat	1:600	120 min	Vector Labs	BA-9010-1.5
Anti-Rabbit (Biotin-SP)	Donkey	1:600	120 min	Jackson	711-065-152
Anti-Chicken (alexa488)	Donkey	1:200	120 min	Jackson	703-545-155
Anti-Rabbit (alexa555)	Donkey	1:200	120 min	Invitrogen	A-31572
Anti-Mouse (alexa647)	Donkey	1:200	120 min	Invitrogen	A-31571

#### Statistical analysis

The *GraphPad* Prism software was used for analysis. Mann–Whitney test was used for comparison of two groups. For rodent studies, sample size was estimated using GPower 3.1 software. Experimental subjects were not randomized.

## Results

### Whole-body biodistribution of AAV9P31 upon i.v., i.c.v. and intraparenchymal administration in C57BL/6 J mice

**Fig. 1:**
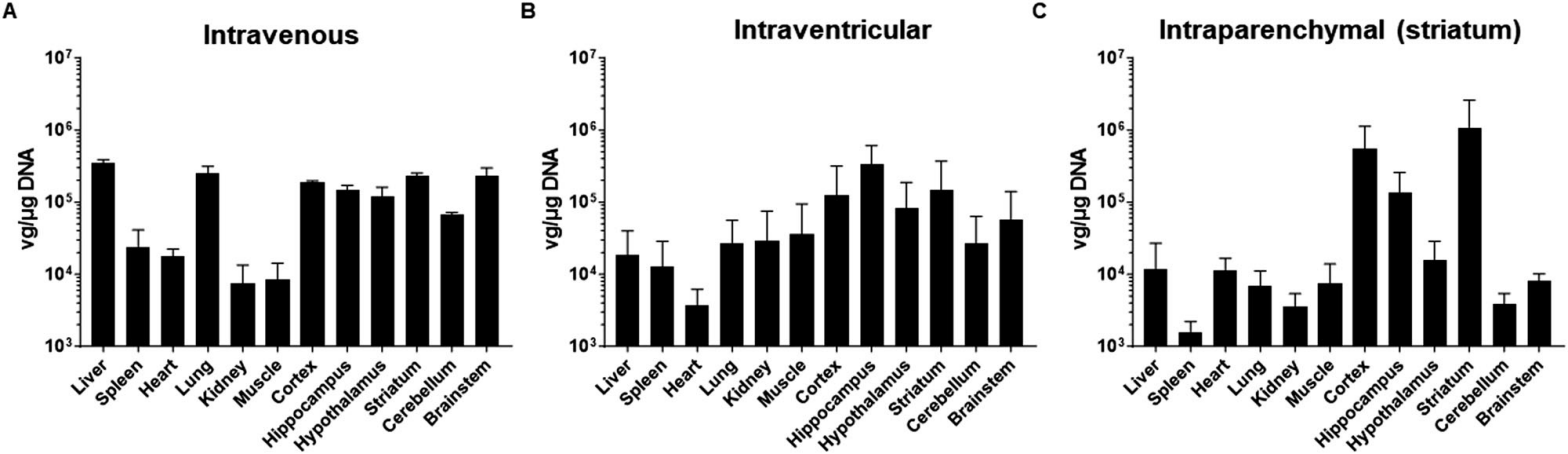
Biodistribution of AAV9P31-CMV-GFP following different routes of administration in C57BL6/J mice. Different groups of 7 weeks-old C57BL6/J mice (3 females and 3 males) received the AAV9P31-CMV-GFP vector by i.v. (retro-orbital) injection at 2 × 10^13^ vg/kg (**A**), or by i.c.v. injection at 1.2 × 10^11^ vg (**B**), or by unilateral intrastriatal injection at 6 × 10^10^ vg (**C**). One month later, mice were sacrificed and viral genomes were quantified in the indicated CNS structures and peripheral organs by qPCR.

**Fig. 2:**
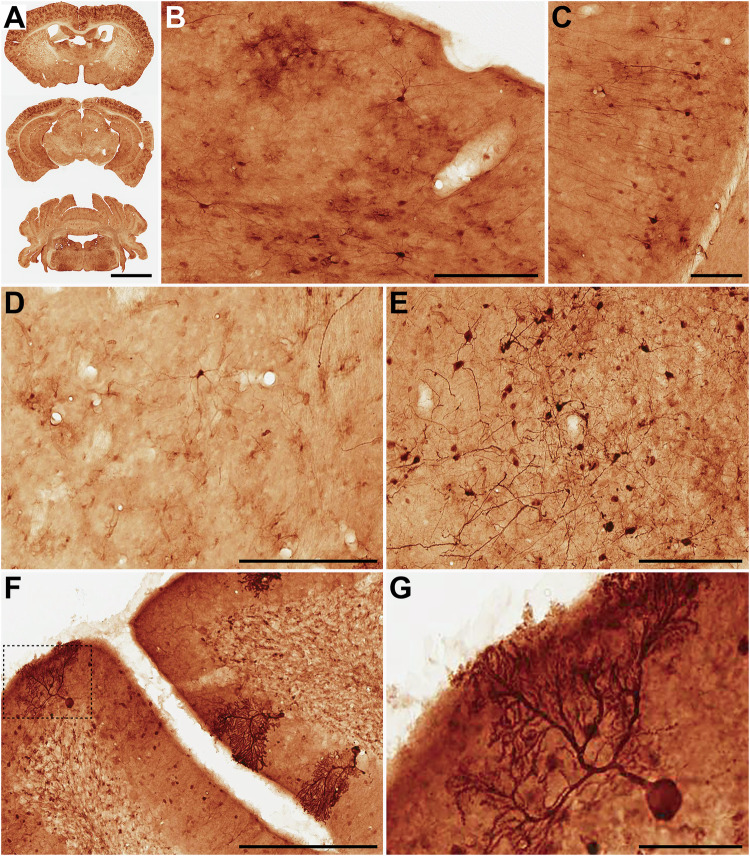
Detection of transduced cells upon administration of AAV9P31-CMV-GFP following different routes in C57BL6/J mice. Different groups of 7 weeks-old C57BL6/J mice (3 females and 3 males) received the AAV9P31-CMV-GFP vector by i.v. (retro-orbital) injection at 2 × 10^13^ vg/kg (**A**), or by i.c.v. injection at 1.2 × 10^11^ vg (**B**), or by unilateral intrastriatal injection at 6 × 10^10^ vg (**C**). One month later, mice were sacrificed and tissue samples were processed for detection of GFP by immunohistochemistry. Pictures show representative images of the indicated tissues. **A** Low-power photomicrographs taken from three different rostrocaudal levels of the CNS illustrating the brain-wide expression of GFP. **B** At the level of the cerebral cortex, a predominant transduction of astrocytes was constantly observed, together with a more moderate expression being found in both pyramidal and non-pyramidal neurons. Image taken from the primary somatosensory cortex. **C** Cellular phenotypes expressing GFP in the hippocampal formation are limited to neurons (image taken from the CA1 field). **D** The striatum is the brain territory showing the weakest GFP expression, mainly comprising scattered astrocytes and medium-sized spiny neurons. **E** Basal forebrain neurons were strongly transduced with GFP. **F**, **G** Different types of neurons in the cerebellum were transduced with GFP, comprising neurons in the molecular, Purkinje and granular layers of the cerebellar cortex. Purkinje neurons were randomly transduced, most often showing a Golgi-like stain (inset in G). Scale bars are 3.0 mm in (**A**); 200 µm in (**B**, **D,** **E**); 100 µm in (**C**); 250 µm in (**F**) and 50 µm in (**G**).

The AAV9P31-CMV-GFP vector was administered to different groups of 7 weeks-old C57BL/6 J mice using the following routes of administration: i.v. (4 × 10^11^ vg, equivalent to 2 × 10^13^ vg/kg); i.c.v. (1.2 × 10^11^ vg, unilateral), and intraparenchymal (6 × 10^10^ vg, intrastriatal, unilateral). One month later, mice were sacrificed and vector genomes were quantified in tissue samples by qPCR. We confirmed the efficient access of the vector to the brain and liver upon i.v. administration, as previously reported [21]. In addition, we found that the lung is also a preferential site for accumulation of AAV9P31 genomes (Fig. 1A). Detailed analysis of different brain regions revealed a relatively homogeneous infection of all structures. In contrast, direct injection into the striatum led to high viral genome content in this location, together with the parieto-temporal cortex in the tract of injection. The access to other brain regions distant from the injection site such as brainstem and cerebellum was significantly lower (Fig. 1C). As expected, the spread of the vector to peripheral organs was low. The i.c.v. administration showed an intermediate phenotype, with clear accumulation of genomes in the brain, especially hippocampus, but relatively lower content in cerebellum and brainstem (Fig. 1B). However, no statistically significant differences were observed among brain structures because the inter-individual variability was relatively high. Immunohistochemical analysis of brain sections confirmed the pattern of transgene expression (GFP) in the brain of mice injected systemically with AAV9P31-CMV-GFP (Fig. 2). The efficacy of hepatocyte transduction following this route of administration was moderate but clearly higher than the i.c.v. route (supplementary fig. 1B, C). Only the intraparenchymal route showed no systemic exposure to the vector (supplementary fig. 1D).

**Fig. 3:**
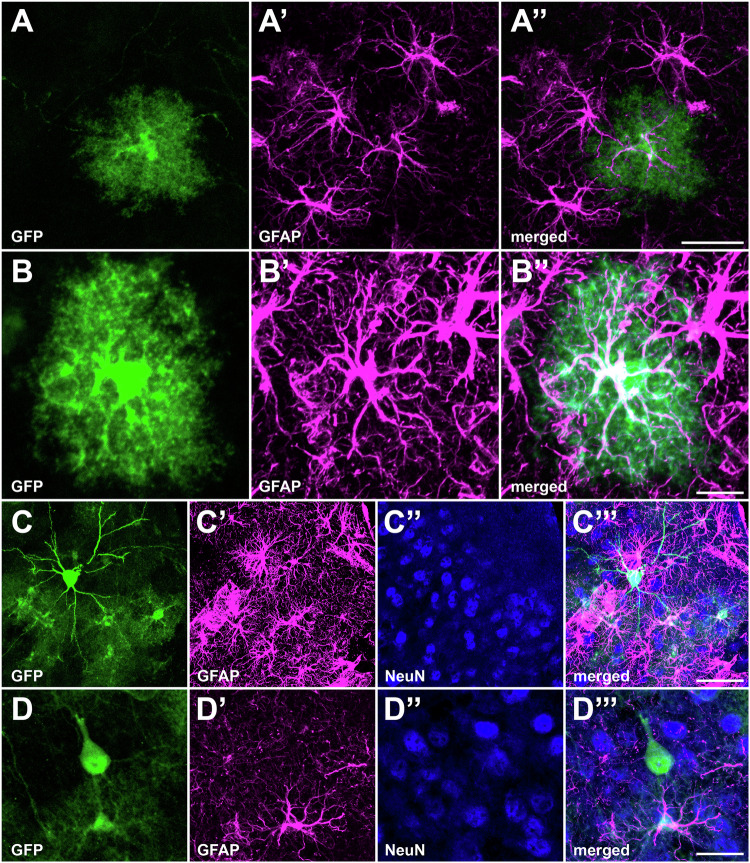
Cellular phenotypes expressing GFP in the cerebral cortex upon i.v. delivery of AAV9P31-CMV-GFP in mice. C57BL6/J mice (3 females and 3 males) received the AAV9P31-CMV-GFP vector by i.v. (retro-orbital) injection at 2 × 10^13^ vg/kg. One month later, mice were sacrificed and brain samples were processed for immunofluorescence using antibodies against GFP (green) and the indicated cell markers: NeuN for neurons (blue) and GFAP for astrocytes (purple). **A-A”** A random transduction of astrocytes was found throughout the cerebral cortex. **B-B”** Expression of GFP spreads within the whole cytoplasm of transduced astrocytes, even reaching the most distal and thinnest astrocytic processes. **C**, **D”** Pyramidal neurons were also randomly transduced, sometimes showing Golgi-like morphologies. A representative example of a GFP^+^ layer II-III pyramidal neuron and surrounding astrocytes is shown in (**C**-**C**”’), whereas (**D**-**D**”’) illustrate a GFP^+^ layer V pyramidal neuron as well as a neighboring transduced astrocyte. Scale bars are 20 μm in (**A**-**A**” & **D**-**D**”); 10 μm in (**B**-**B**”) and 40 μm in (**C**-**C**”).

**Fig. 4:**
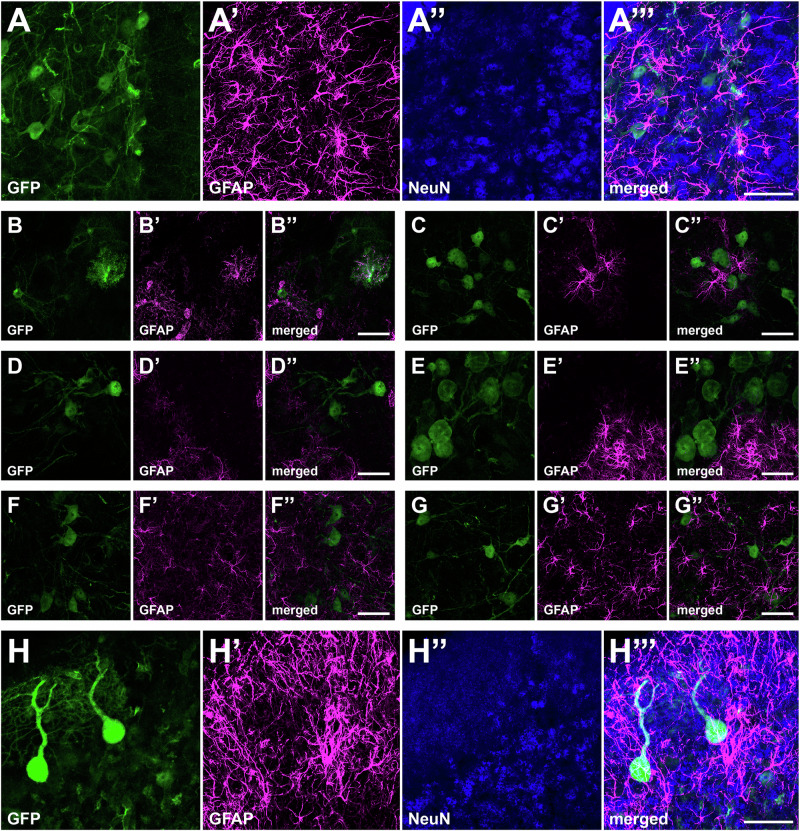
Cellular phenotypes expressing GFP in subcortical locations upon i.v. delivery of AAV9P31-GFP in mice. C57BL6/J mice (3 females and 3 males) received the AAV9P31-CMV-GFP vector by i.v. (retro-orbital) injection at 2 × 10^13^ vg/kg. One month later, mice were sacrificed and brain samples were processed for immunofluorescence using antibodies against GFP (green) and the indicated cell markers: NeuN for neurons (blue) and GFAP for astrocytes (purple). In all analyzed subcortical territories, neurons were found to be the only cellular phenotype transduced with GFP, as shown for the hippocampal formation (**A-A”’**; image taken from the CA1 field); lateral hypothalamic nuclei (**C-C”’**), substantia nigra pars compacta (**D-D”’**), locus coeruleus (**E-E”’**), as well as in motor and sensory brainstem nuclei such as the facial nerve (**F-F”’**), and inferior olive (**G-G”’**) nuclei, respectively. The same pattern of exclusive neuronal transduction was found in the cerebellar cortex, where only Purkinje and granule neurons were GFP^+^ (**H-H”’**). The striatum was found to be the only subcortical structure with few GFP^+^ astrocytes (**B-B”**). Scale bars are 40 μm in all panels.

In order to determine the tropism of the vector for different cell populations in the CNS, we performed multiple immunofluorescent stains in brain sections from C57BL/6 J mice treated with AAV9P31-CMV-GFP. Co-staining was performed with GFP for AAV transduction and specific markers for neurons (NeuN), astroglia (GFAP), and microglia (Iba1). Although the conducted immunoperoxidase stains upon i.v. delivery of AAV9P31-CMV-GFP showed a widespread transduction throughout the entire brain, immunofluorescent stains revealed different patterns of cellular transduction between different brain regions. In this regard, both the cerebral cortex and the striatum showed a preferential and random transduction of astrocytes, as well as projection neurons (Fig. 3). Cortical astrocytes were transduced very strongly, with GFP expression extending over the thinnest astrocytic processes (Fig. 3A, B). By contrast, neurons were found to be the only cell type expressing GFP within any examined subcortical locations such as the hippocampus, amygdaloid complex, thalamus, hypothalamus, mesencephalon, sensory and motor-related nuclei of brainstem and cerebellum (Fig. 4). Furthermore, GFP expression was never found in microglial cells regardless the brain territory under scrutiny (Supplementary Figs. 2, 3).

### Spatial and temporal analysis of transgene expression upon i.v. administration of AAV9P31

**Fig. 5:**
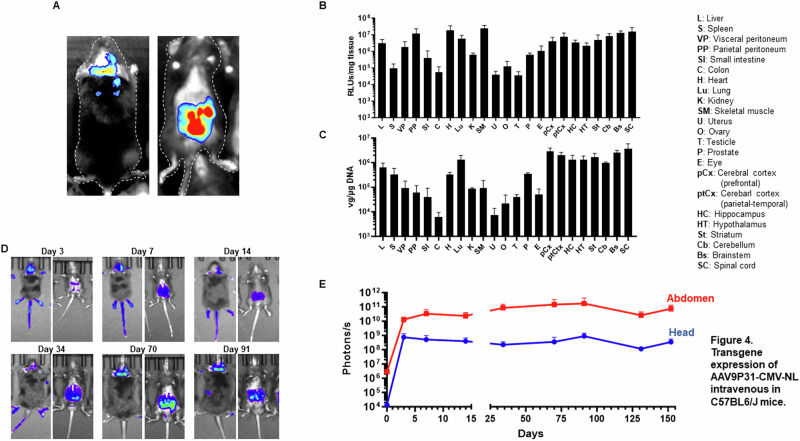
Biodistribution and kinetics of transgene expression in C57BL/6 J mice treated with i.v. injection of AAV9P31-CMV-NL. **A–C** Seven weeks-old C57BL/6 J mice (3 females and 3 males) received the AAV9P31-CMV-NL vector by i.v. (retro-orbital) injection at 2 × 10^13^ vg/kg. One month later, mice received an intraperitoneal injection of the NanoLuc substrate (furimazine) and light emission was visualized in a luminometer (**A**). The pictures show a representative image of the dorsal and ventral views. Mice were then sacrificed for dissection of different CNS structures and peripheral organs. One portion of the tissue samples was used for quantification of luciferase activity ex vivo (**B**), and other portion was processed DNA isolation and quantification of viral genomes by qPCR (**C**). The same dose of vector and route of administration was used to treat 2 weeks-old C57BL/6 J mice (*n* = 6). Longitudinal detection (**D**) and quantification (**E**) of NanoLuc expression was performed by BLI at the indicated times after vector administration. In the BLI pictures, maximal and minimal light emission are represented by red and blue pseudocolors, respectively. Quantification corresponds to total light emission (photons/s) from ROIs located in the head and abdominal areas.

In order to validate the results obtained with the AAV9P31-GFP vector and to gain insight into the kinetics of transgene expression, we employed the NanoLuc-expressing vector AAV9P31-CMV-NL. In the first experiment, 7 weeks-old C57BL/6 J mice received the vector by i.v. injection (2 × 10^13^ vg/kg). One month later, mice were treated with an intraperitoneal injection of the substrate (furimazine), and light emission was detected by a luminometer in live animals (Fig. 5A). A strong signal was detected in the brain area and the abdomen. However, spatial definition of BLI is low, and light can be quenched by overlapping tissues such as bone or hair. In order to define which organs and brain regions were responsible for light emission, mice were sacrificed and luciferase activity was measured in tissue extracts (Fig. 5B). The results confirm widespread and relatively homogeneous transgene expression across all CNS structures, including cerebellum, brainstem and spinal cord. Interestingly, peripheral organs expressing high levels of NanoLuc were not only liver and lung, but also peritoneum, heart and skeletal muscle. Only residual expression was observed in the colon, gonads and uterus. In contrast, the prostate showed moderate levels, similar to the eyes. In general, these results are consistent with the detection of viral genomes in the same organs (Fig. 5C), although the ratio between NanoLuc expression and viral genomes is variable among tissues (Supplementary Fig. 4). In order to study the kinetics of transgene expression, two weeks-old C57BL/6 J mice received the AAV9P31 vector by i.v. injection, and light emission was monitored for up to 5 months (Fig. 5D, E). We found strong signals as early as 72 h after vector administration both in the head and abdominal regions. After a moderate decay during the first 3 weeks, transgene expression remained stable until the end of the experiment in both regions.

### Biodistribution and transgene expression of AAV9P31 in Balb/c mice upon i.v. administration of AAV9P31

**Fig. 6:**
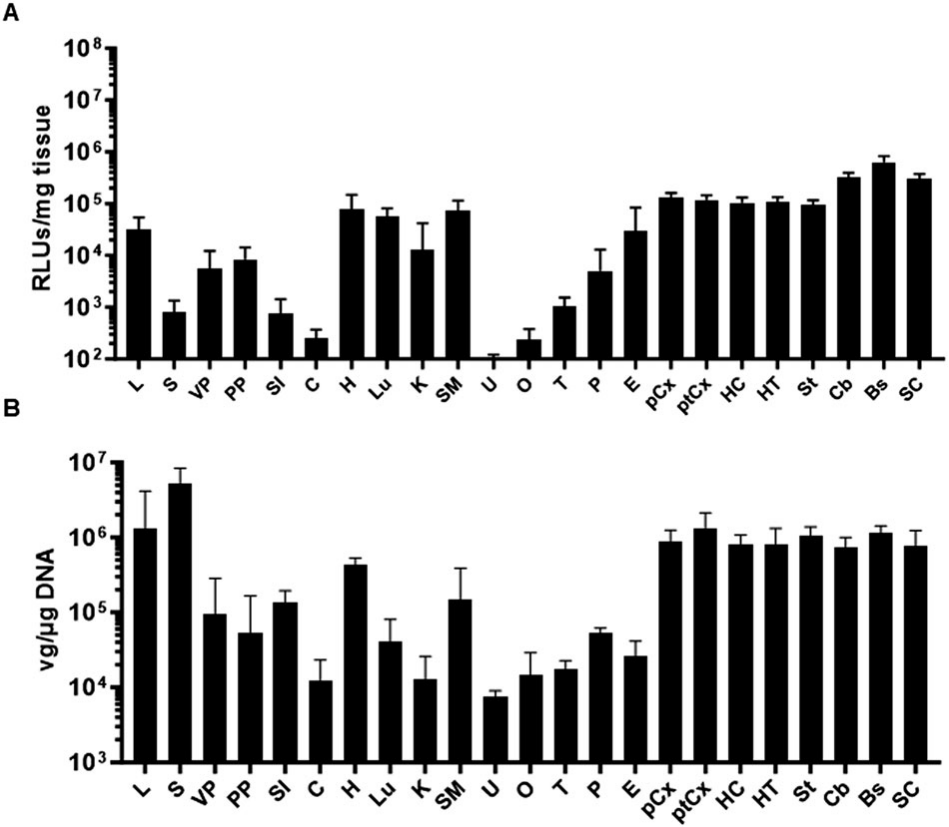
Biodistribution and transgene expression in Balb/c mice treated with i.v. injection of AAV9P31-CMV-NL. Seven weeks-old Balb/c mice (3 females and 3 males) received the AAV9P31-CMV-NL vector by i.v. (retro-orbital) injection at 2 × 10^13^ vg/kg. One month later, mice were sacrificed for dissection of different CNS structures and peripheral organs. One portion of the tissue samples was used for quantification of luciferase activity ex vivo (**A**), and other portion was processed DNA isolation and quantification of viral genomes by qPCR (**B**).

Next, we studied if the ability of AAVP31 to cross the BBB was maintained in other commonly used mouse strains such as Balb/c mice. To this end, the AAV9P31-CMV-NL vector was injected i.v. in this strain (2 × 10^13^ vg/kg), and the animals were sacrificed one month later to quantify vector genomes and luciferase activity in different brain structures and peripheral organs. The pattern of transgene expression (NanoLuc) in Balb/c mice was similar to C57BL/6 J (Fig. 6A), in agreement with the conservation of CA-IV across different mouse strains [25], supplementary fig. 5. The distribution of genomes was also common, with some exceptions such as higher vector genomes in the spleen and lower in the lung of Balb/c mice compared with C57BL/6 J (Fig. 6B). Efficient and homogeneous transduction of brain structures was confirmed. Interestingly, Balb/c mice presented lower NanoLuc expression levels relative to the vector genome content in all tissues studied, in comparison with C57BL/6 J (Supplementary Fig. 4).

### Biodistribution of AAV9P31 in rats and NHPs

**Fig. 7:**
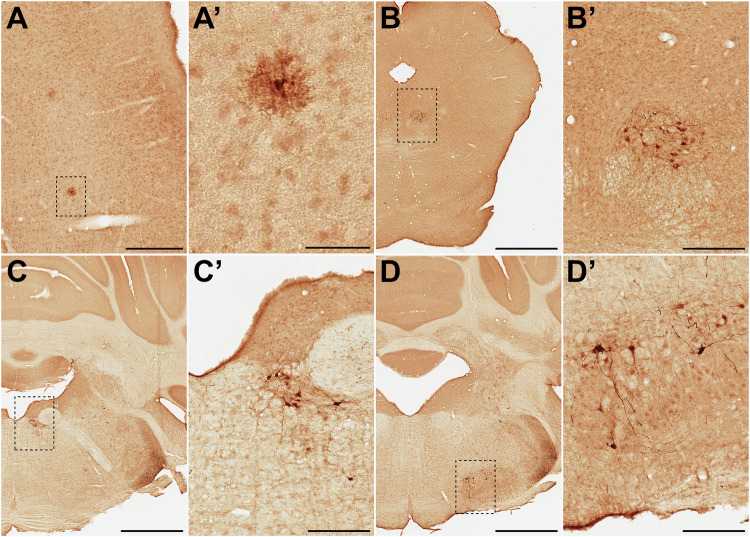
Immunoperoxidase detection of GFP transduced cells upon i.v. delivery of AAV9P31-CMV-GFP in rats. Seven weeks-old rats (one female and one male) received the AAV9P31-CMV-GFP vector by i.v. (retro-orbital) injection at 2 × 10^13^ vg/kg. One month later, rats were sacrificed and tissue samples were processed for detection of GFP by immunohistochemistry. **A-A’** Low-power photomicrograph and inset taken from the primary somatosensory cortex showing just a few, scattered astrocytes expressing GFP across the whole rostrocaudal extent of the cerebral cortex. **B–D’** GFP^+^ neurons were unexpectedly found in different locations of the caudal mesencephalon and brainstem such as the dorsal raphe nucleus (**B-B’**), the abducens nucleus (**C-C’**) and the inferior olive (**D-D’**). GFP^+^ neurons were only found in these discrete locations of the rat brain, together with few scattered neurons in different brainstem nuclei. Scale bars are 100 µm in panel (**A**); 25 µm in (**B**’); 1000 µm in (**B**, **D**, **E**); and 200 µm in (**B**’, **C**’, **D**’).

**Fig. 8:**
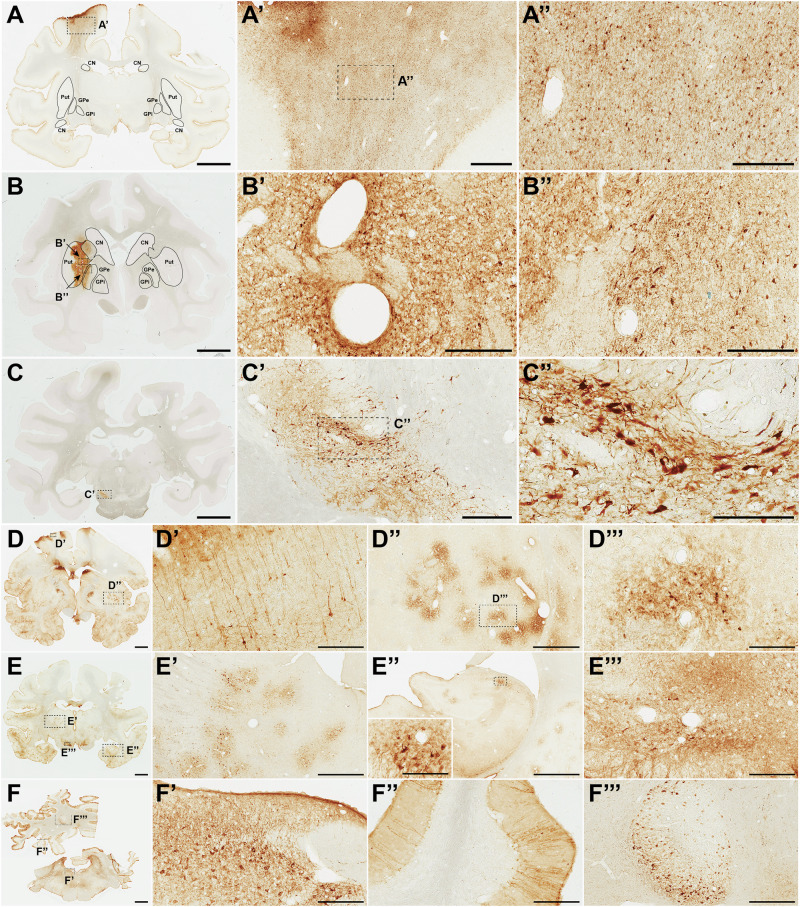
Immunohistochemical analysis of transgene expression in tissues from macaques treated with AAV9P31 vectors by different routes. Macaca fascicularis (*n* = 2, male and female) were treated simultaneously with vectors AAV9P31-CMV-GFP (2 × 10^13^ vg/kg), AAV9P31-CMV-tdT (4 × 10^13^ vg) and AAV9P31-CMV-NL (2 × 10^11^ vg) following i.v., i.c.v. and intraparenchymal injections (substantia nigra), respectively. Animals were sacrificed one month after injection, and the expression of the respective transgenes was analyzed by IHQ. In the case of NanoLuc (AAV9P31-CMV-NL), the antibody was directed against the N-terminal HA tag fused to the protein. **A-A”** The systemic delivery of AAV9P31-CMV-GFP resulted in an almost complete lack of CNS transduction. GFP^+^ astrocytes were only found in the white matter area located above the putamen nucleus through the tract of the intraputaminal delivery of NL (HA-tagged) vector. For orientation purposes, the boundaries of the caudate and putamen nuclei, as well as both segments of the globus pallidus are delineated. **B**, **C** The intraparenchymal delivery resulted in local HA^+^ neurons and astrocytes at the level of the injected putamen nucleus (**B’**). AAV9P31 shared similar retrograde spreading properties of the native AAV9 capsid, therefore leading to transduction of GPe neurons projecting to the putamen (HA^+^ arkypallidal neurons; **B”**), as well as neurons located in the left substantia nigra pars compacta (**C’-C”**) innervating the left putamen nucleus through the nigrostriatal pathway. **D–F** Widespread neuronal transduction in the cerebral cortex, subcortical structures and the cerebellum upon i.c.v. administration of AAV9P31-CMV-tdT. **D’** tdT^+^ pyramidal neurons from upper and lower cortical layers are organized in a columnar random pattern. **D”D”’** Patchy distribution of tdT^+^ neurons in the putamen nucleus, more evident in ventrolateral putaminal areas. **E’** A similar patchy pattern of distribution for tdT^+^ neurons was observed in the thalamus. **E”** A moderate neuronal transduction was found in the hippocampal formation, with a higher number of tdT^+^ neurons in the CA3 field (inset in **E”**). **E”’** tdT^+^ neurons in deep brain structures such as the substantia nigra pars compacta. **F** tdT^+^ structures observed in the brainstem and cerebellum, including neurons within the abducens nucleus in the brainstem (**F’**), climbing fibers in the cerebellum (**F”**), as well as neurons with strong tdT labeling in deep cerebellar nuclei (**F”’**). Abbreviations: caudate nucleus (CN), putamen nucleus (Put), internal division of the globus pallidus (GPi), external division of the globus pallidus (GPe). Scale bars are 3 mm in (**A**–**C**, **F**); 4 mm in (**D**, **E**); 1 mm in (**A**’, **D**”); 300 µm in (**A**”, **B**’, **B**”, **D**’, **E**’, **F**’, **F**”); 500 µm in (**C**’); 200 µm in (**C**”); 150 µm in (**D**”’, **E**”’) & inset in (**E**”); 1500 µm in (**E**”) and 600 µm in (**F**”’).

After characterization of AAV9P31 in the most widely used mouse strains, we investigated to what extent this capsid can bypass the BBB in other species. Initial experiments were carried out by i.v. injection of the AAV9P31-CMV-GFP vector into adult (7 weeks-old) Sprague Dawley rats (2x10^13^ vg/kg). Immunohistochemical analysis of GFP in rat brains performed one month after vector injection revealed no transgene expression in most of the brain territories examined, despite moderate transduction in the liver (Supplementary Fig. 1E). However, neurons transduced with GFP were constantly found in several different nuclei in the brainstem (Fig. 7), therefore suggesting an unexpected and enriched distribution of the targeted receptor specific for the brainstem. For the analysis of AAV9P31 capsid in NHPs, we compared the three routes of administration tested in C57BL/6 J mice. In order to minimize the use of animals and to best compare different administration routes, the same macaques (*n* = 2) were injected with vectors expressing GFP, tdT and NanoLuc following i.v., i.c.v. and intraparenchymal injections, respectively. The i.v. dose was maintained as 2 × 10^13^ vg/kg. For the i.c.v. route, animals received a total of 4 × 10^13^ vg in bilateral injections (2x10^13^ vg in 1 ml/ventricle). The intraparenchymal injection consisted of 2 × 10^11^ vg in a volume of 5 µl injected unilaterally in the left putamen nucleus. Animals were sacrificed one month after injection, and the expression of the respective transgenes was analyzed by immunoperoxidase stains. In the case of NanoLuc, the antibody was directed against the N-terminal HA tag fused to the protein. No meaningful GFP expression was observed in the brain of NHPs injected i.v. with the AAV9P31-CMV-GFP vector, whereas positive hepatocytes were readily detected in the liver (supplementary fig. 1F). GFP^+^ cells were only found through the injection tract from the insertion of the needle used for intraputaminal deliveries and thus leading to a mechanical disruption of the BBB in this location (Fig. 8A), therefore suggesting that the AAV9P31 capsid was unable to bypass the intact BBB in NHPs upon systemic delivery. That said, HA^+^ cells were detected in the injected putamen nucleus (Fig. 8B), indicating the ability of the vector to transduce neurons in the macaque brain following intraparenchymal deliveries. Moreover, the AAV9P31 capsid variant maintained the well-known retrograde spreading properties of the native AAV9 capsid and therefore external division of the globus pallidus (GPe) neurons projecting to the putamen (arkypallidal neurons) also became transduced (Fig. 8B”). Similarly, neurons located in the left substantia nigra pars compacta innervating the left putamen nucleus through the nigrostriatal pathway were also retrogradely transduced upon intraparenchymal delivery of the vector (Fig. 8C’-C”). Finally, the i.c.v. administration of AAV9P31-CMV-tdT led to a widespread neuronal transduction in the cerebral cortex, subcortical structures and the cerebellum (Fig. 8D–F”’), likely mimicking the ability of the native AAV9 capsid to bypass the ependymal brain barrier instead of reflecting a receptor-mediated transport. At the cortical level, pyramidal neurons from upper and lower cortical layers became transduced. Neurons positive for tdT are organized in a columnar random pattern (Fig. 8D, D’). Moreover, a patchy distribution of neurons was noticed in the putamen nucleus, more evident in ventrolateral putaminal areas (Fig. 8D”, D”’). A similar patchy distribution of transduced neurons was found in the thalamus (Fig. 8E, E’). A moderate neuronal transduction was found in the hippocampal formation (Fig. 8E”), with a higher number of tdT^+^ neurons being found in the CA3 field (inset in E”). Neurons expressing tdT were also found in deep brain structures such as the substantia nigra pars compacta (Fig. 8E”’). Regarding the brainstem, the most abundant neuronal transduction was found in peri-ventricular locations such as the abducens nucleus (Fig. 8F’). At the level of the cerebellar cortex, climbing fibers were the only structures expressing tdT (Fig. 8F”), whereas neurons located in deep cerebellar nuclei were strongly transduced (Fig. 8F”’).

## Discussion

The development of methods to screen AAV capsid libraries in vivo is opening new opportunities to find vectors with the ability to transduce the CNS upon systemic administration [17, 21]. This route is not only more convenient and less invasive than current options, but also it allows wide and homogeneous coverage of the entire brain. This property is instrumental for addressing a variety of genetic encephalopathies, as well as other potential candidate diseases characterized by a widespread distribution of the related pathology such as autism, schizophrenia or Alzheimer’s disease, among others. The downside of systemic administration is the sequestration of vectors in organs such as liver, spleen and lung, and the potential side effects due to ectopic expression of the transgene [26]. Detargeting strategies based on capsid design are challenging, but some progress has been done [27, 28]. Inhibition of transgene expression in unwanted organs can be obtained by insertion of specific miRNA target sequences in the 3’UTR [29]. Peptide-displaying capsid libraries are agnostic methods, but they have contributed to generate valuable knowledge to increase the relevance of results. Key achievements include the identification of exposed capsid domains that are permissive for peptide insertion [16], and the discovery of the target receptors responsible for trans-cytosis through brain endothelial cells [20, 22, 30]. It has become evident that the identification of a given capsid by in vivo screening in one species cannot be extrapolated to others [19, 22]. Once the receptor of the displayed peptide is known, it becomes easier to predict if the behavior will be conserved across strains or species. Still, in vivo validation is needed because many factors may influence the final outcomes. The results presented in this work reinforce the notion that CA-IV is the receptor used by AAV9P31 to cross the BBB [22]. The CA-IV region involved in this interaction is identical between C57BL/6 J and Balb/c mouse strains [25], whereas conservation is low in primates. The rat presents an intermediate situation, and we show here that this degree of homology is not sufficient to maintain a functional binding to AAV9P31. However, the obtained unexpected transduction in the brainstem of rats injected i.v. with AAV9P31-CMV-GFP suggested an uneven distribution of the CA-IV receptor in the BBB of rats, not reported previously. Moreover, the observed transduction following the i.c.v. delivery of AAV9P31-CMV-tdT in non-human primates is likely mimicking similar properties reported for the native AAV9 capsid regarding its ability to penetrate the ependymal barrier [31] instead of being mediated by interaction with the CA-IV receptor. Assuming that the scope of this capsid variant may be limited to mouse models, we focused our efforts in the characterization of this species. Biodistribution and transgene expression upon systemic administration shows a reasonable correlation with the pattern of expression of CA-IV, which is abundant in the brain, lung, and gastrointestinal tract (https://www.gtexportal.org/). We observed higher vector genome content and transgene expression in peritoneum than in colon or small intestine, which was not previously described. We confirmed the previous observation of high transgene expression in the heart of C57BL/6 J, despite moderate levels of vector genomes [21]. In our case, NanoLuc expression was also elevated in skeletal muscle. In general, C57BL/6 J mice presented higher transgene expression relative to vector genomes in all tissues analyzed, a phenomenon deserving further in-depth studies. The kinetics of BLI revealed rapid and stable NanoLuc expression when the vector was i.v. injected in 2 weeks-old infantile mice, which is important for gene therapy of genetic encephalopathies.

The search for the optimal peptide-receptor pair for crossing the human BBB is still ongoing [32], and it will require a combination of tests in cell culture human BBB models, in vivo experiments in NHPs, and clinical trials before having a chance to appoint a robust candidate. During the revision of this manuscript, the first reports of AAV capsids with the ability to target conserved receptors across species and to bypass the BBB in rodents and NHPs have been released [33]. Furthermore, another capsid designed for binding to the human transferrin receptor showed widespread brain transduction in a humanized mouse model [34]. The availability and clinical relevance of these variants will be determined in the near future. In the meantime, the AAV9P31 capsid can be very useful to perform basic research and to evaluate CNS-directed therapies. Considering the versatility of AAV capsids, it is realistic to assume that a pre-clinical vector can be rapidly adapted for clinical use by swapping the mouse and human peptides, although it should be acknowledged that these would be two different products. Moreover, the use of the “open access” AAV9P31 capsid variant may play an instrumental role when applied to transgenic mouse models of neurodegenerative disorders such as Alzheimer’s and Parkinson’s disease, these models currently reproducing the underlying neuropathological signatures only to a limited extent [35]. However, a broad perspective should be applied to determine the translational value of our results. When compared to delivery routes such as intra-CSF and intraparenchymal deliveries, the systemic administration of a given AAV candidate has its own advantages and disadvantages. On one hand, systemic deliveries are minimally invasive and do not require stereotaxic surgery (needed for regional and local administrations). On the other hand, even assuming an efficient transition through the BBB, high vector loads will be needed; off-target effects are very likely, and pre-testing for neutralizing antibodies is a pre-requisite. Furthermore, capsid modifications may change the tropism of the vector for different cell populations in the brain, introducing an additional layer of uncertainty for clinical application. In summary, although current generations of BBB-penetrant AAV capsid variants are collectively viewed as steps in the right direction, there still is a large room for improvement, and careful clinical investigation should be performed to determine which capsid variants and routes of administration are best suited for a particular human disease.

In conclusion, our results confirm the AAV9P31 variant as a suitable platform for preclinical CNS research, with unprecedented possibilities for both disease modeling as well as for certain therapeutic applications.

## Supplementary information

Suppl Figures (collated)

## Data Availability

The original contributions presented in the study are included in the article/supplementary material, further inquiries can be directed to the corresponding authors. Further information and request for resources and reagents should be directed and will be fulfilled by the corresponding authors RH-A (rubenh@unav.es) and JLL (jlanciego@unav.es).

## References

[1] Padmakumar S, Souza AD, Parayath NN, Bleier BS, Amiji MM. Nucleic acid therapies for CNS diseases: Pathophysiology, targets, barriers, and delivery strategies. J Control Release [Internet]. 2022;352:121–45. 10.1016/j.jconrel.2022.10.018. 36252748 10.1016/j.jconrel.2022.10.018

[2] Fu H, Mccarty DM. Crossing the blood – brain-barrier with viral vectors. Curr Opin Virol. 2016;21:87–92. 27591676 10.1016/j.coviro.2016.08.006

[3] Seko I, Şahin A, Tonbul H, Çapan Y. Brain - targeted nanoparticles to overcome the blood - brain barrier. J Pharm Technol. 2020;1:25–39.

[4] Chandran J, Chowdhury EA, Perkinton M, Jamier T. Assessment of AAV9 distribution and transduction in rats after administration through Intrastriatal, Intracisterna magna and Lumbar Intrathecal routes. Gene Ther. 2023;30:132–41. 35637286 10.1038/s41434-022-00346-1

[5] Galvan A, Petkau TL, Hill AM, Korecki AJ, Lu G, Choi D, et al. Intracerebroventricular administration of AAV9-PHP. B SYN1-EmGFP induces widespread transgene expression in the Mouse and Monkey central nervous system. Hum Gene Ther 2021;32:599–615. 33860682 10.1089/hum.2020.301 PMC8236560

[6] Kumagai S, Nakajima T, Shimazaki K, Kakiuchi T, Harada N, Ohba H, et al. Early distribution of 18 F-labeled AAV9 vectors in the cerebrospinal fluid after intracerebroventricular or intracisternal magna infusion in non-human primates. J Gene Med. 2023;25:e3457. 36278965 10.1002/jgm.3457

[7] Hereñú CB, Sonntag WE, Morel GR, Portiansky EL, Goya RG. The ependymal route for insulin-like growth factor-1 gene therapy in the brain. NSC [Internet] 2009;163:442–7. 10.1016/j.neuroscience.2009.06.024. 10.1016/j.neuroscience.2009.06.024 PMC2740751 19531373

[8] Van Putten EHP, Kleijn A, Van Beusechem VW, Noske D, Lamers CHJ, De Goede AL, et al. Convection enhanced delivery of the oncolytic adenovirus delta24-RGD in patients with recurrent GBM: a phase I clinical trial including correlative studies. Clin Cancer Res. 2022;28:1572–85. 35176144 10.1158/1078-0432.CCR-21-3324 PMC9365362

[9] Mora-Jimenez L, Valencia M, Sanchez-Carpintero R, Tønnesen J, Fadila S, Rubinstein M, et al. Transfer of SCN1A to the brain of adolescent mouse model of Dravet syndrome improves epileptic, motor, and behavioral manifestations. Mol Ther Nucleic Acids [Internet]. 2021;25:585–602. 10.1016/j.omtn.2021.08.003. 34589280 10.1016/j.omtn.2021.08.003 PMC8463324

[10] McFarland NR, Lee JS, Hyman BT, McLean P. Comparison of transduction efficiency of recombinant AAV serotypes 1, 2, 5, and 8 in the rat nigrostriatal system. J Neurochem. 2009;109:838–45. 19250335 10.1111/j.1471-4159.2009.06010.x PMC2698947

[11] Kang L, Jin S, Wang J, Lv Z, Xin C, Tan C, et al. AAV vectors applied to the treatment of CNS disorders: clinical status and challenges. J Control Release [Internet]. 2023;355:458–73. 10.1016/j.jconrel.2023.01.067. 36736907 10.1016/j.jconrel.2023.01.067

[12] Liguore WA, Domire JS, Button D, Wang Y, Dufour BD, Srinivasan S, et al. AAV-PHP.B administration results in a differential pattern of CNS biodistribution in non-human primates compared with mice. Mol Ther [Internet]. 2019;27:2018–37. 10.1016/j.ymthe.2019.07.017. 31420242 10.1016/j.ymthe.2019.07.017 PMC6838922

[13] Tai C, Lee N, Chien Y, Byrne BJ, Muramatsu S, Tseng S, et al. Long-term efficacy and safety of eladocagene exuparvovec in patients with AADC deficiency. Mol Ther [Internet]. 2022;30:509–18. 10.1016/j.ymthe.2021.11.005. 34763085 10.1016/j.ymthe.2021.11.005 PMC8822132

[14] Symonds JD, Zuberi SM, Stewart K, Mclellan A, Regan MO, Macleod S, et al. Incidence and phenotypes of childhood-onset genetic epilepsies: a prospective population-based national cohort. Brain. 2019;142:2303–18. 31302675 10.1093/brain/awz195 PMC6658850

[15] Challis RC, Ravindra Kumar S, Chen X, Goertsen D, Coughlin GM, Hori AM, et al. Adeno-associated virus toolkit to target diverse brain cells. Annu Rev Neurosci. 2022;45:447–69. 35440143 10.1146/annurev-neuro-111020-100834

[16] Börner K, Kienle E, Huang L, Weinmann J, Sacher A, Bayer P, et al. Pre-arrayed Pan-AAV peptide display libraries for rapid single-round screening. Mol Ther. 2020;28:1016–32. 32105604 10.1016/j.ymthe.2020.02.009 PMC7132618

[17] Deverman BE, Pravdo PL, Simpson BP, Kumar SR, Chan KY, Banerjee A, et al. Cre-dependent selection yields AAV variants for widespread gene transfer to the adult brain. Nat Biotechnol. 2016;34:204–9. 26829320 10.1038/nbt.3440 PMC5088052

[18] Chan KY, Jang MJ, Yoo BB, Greenbaum A, Ravi N, Wu W, et al. Engineered AAVs for efficient noninvasive gene delivery to the central and peripheral nervous systems. Nat Neurosci. 2017;20:1172–81. 28671695 10.1038/nn.4593 PMC5529245

[19] Matsuzaki Y. Neurotropic properties of AAV-PHP. B are shared among diverse inbred strains of mice. Mol Ther. 2019;27:700–4. 30842039 10.1016/j.ymthe.2019.02.016 PMC6453512

[20] Hordeaux J, Yuan Y, Clark PM, Wang Q, Martino RA, Sims JJ, et al. The GPI-linked protein LY6A drives AAV-PHP. B transport across the blood-brain barrier. Mol Ther [Internet]. 2019;27:912–21. 10.1016/j.ymthe.2019.02.013. Available from 30819613 10.1016/j.ymthe.2019.02.013 PMC6520463

[21] Nonnenmacher M, Wang W, Child MA, Ren XQ, Huang C, Ren AZ, et al. Rapid evolution of blood-brain-barrier-penetrating AAVcapsids by RNA-driven biopanning. Mol Ther - Methods Clin Dev [Internet]. 2021;20:366–78. 10.1016/j.omtm.2020.12.006. 10.1016/j.omtm.2020.12.006 PMC7841218 33553485

[22] Shay TF, Sullivan EE, Ding X, Chen X, Kumar SR, Goertsen D, et al. Primate-conserved carbonic anhydrase IV and murine- restricted LY6C1 enable blood-brain barrier crossing by engineered viral vectors. Sci Adv. 2023;9:eadg6618. 37075114 10.1126/sciadv.adg6618 PMC10115422

[23] Piedra Jose, Ontiveros Maria, Miravet Susana, Penalva Cristina, Monfar Merce, Chillon M. Development of a rapid, robust, and universal picogreen-based method to titer adeno-associated vectorsle. Hum Gene Ther Methods. 2015;26:35–42. 25640021 10.1089/hgtb.2014.120

[24] Lanciego JL, Vazquez A. The basal ganglia and thalamus of the long-tailed macaque in stereotaxic coordinates. A template atlas based on coronal, sagittal and horizontal brain sections. Brain Struct Funct. 2012;217:613–66. 22179107 10.1007/s00429-011-0370-5 PMC3322328

[25] Hurt JD, Tu C, Laipis PJ. Isolation and expression of murine carbonic anhydrase IV 1. Protein Expr Purif. 1998;16:7–16. 10.1006/prep.1997.0801 9473451

[26] Whiteley LO. An overview of nonclinical and clinical liver toxicity associated with AAV gene therapy. Toxicol Pathol. 2023;51:400–4. 37772805 10.1177/01926233231201408

[27] Goertsen D, Flytzanis NC, Goeden N, Chuapoco MR, Cummins A, Chen Y, et al. AAV capsid variants with brain-wide transgene expression and decreased liver targeting after intravenous delivery in mouse and marmoset. Nat Neurosci. 2022;25:106–15. 34887588 10.1038/s41593-021-00969-4

[28] Chuapoco MR, Flytzanis NC, Goeden N, Octeau JC, Roxas KM, Chan KY, et al. Adeno-associated viral vectors for functional intravenous gene transfer throughout the non-human primate brain. Nat Nanotechnol. 2023;18:1241–51. 37430038 10.1038/s41565-023-01419-x PMC10575780

[29] Geisler A, Jungmann A, Kurreck J, Poller W, Katus HA, Vetter R, et al. microRNA122-regulated transgene expression increases specificity of cardiac gene transfer upon intravenous delivery of AAV9 vectors. Gene Ther. 2011;18:199–209. 21048795 10.1038/gt.2010.141

[30] Huang Q, Chen AT, Chan KY, Sorensen H, Barry AJ, Azari B, et al. Targeting AAV vectors to the central nervous system by engineering capsid – receptor interactions that enable crossing of the blood – brain barrier. Plos Biol [Internet]. 2023;21:e3002112 10.1371/journal.pbio.3002112. 37467291 10.1371/journal.pbio.3002112 PMC10355383

[31] Samaranch L, Kells AP, Foust KD, Salegio EA, Sebastian WS, Bringas JR, et al. Adeno-associated virus serotype 9 transduction in the central nervous system of nonhuman primates. Hum Gene Ther. 2012;389:382–9. 10.1089/hum.2011.200 PMC3327605 22201473

[32] Chen X, Wolfe DA, Bindu DS, Zhang M, Taskin N, Goertsen D, et al. Functional gene delivery to and across brain vasculature of systemic AAVs with endothelial-speci fi c tropism in rodents and broad tropism in primates. Nat Commun. 2023;14:3345. 37291094 10.1038/s41467-023-38582-7 PMC10250345

[33] Moyer TC, Hoffman BA, Chen W, Shah I, Ren X-Q, Knox T, et al. Highly conserved brain vascular receptor ALPL mediates transport of engineered viral vectors across the blood-brain barrier. BioRxiv. 2024. 10.1101/2024.03.12.584703. 10.1016/j.ymthe.2025.04.046 PMC12461625 40340250

[34] Huang Q, Chan KY, Wu J, Botticello-romero NR, Zheng Q, Lou S, et al. An AAV capsid reprogrammed to bind human transferrin receptor mediates brain-wide gene delivery. Science (80-) 2024;1227:1220–7. 10.1126/science.adm8386 PMC13387621 38753766

[35] Ransohoff RM. All (animal) models (of neurodegeneration) are wrong. Are they also useful? J Exp Med. 2018;215:2955–8. 30459159 10.1084/jem.20182042 PMC6279414

