# Supplementary material for: Characterization of brain transduction capability of a BBB-penetrant AAV vector in mice, rats and macaques reveals differences in expression profiles
Source: Gene Ther. 2024 Jul 22;31(9-10):455–66. doi: 10.1038/s41434-024-00466-w (PMC11399087; doi:10.1038/s41434-024-00466-w)
Supplement: Supplementary file 1 — Suppl Figures (collated) [file 41434_2024_466_MOESM1_ESM.pdf]

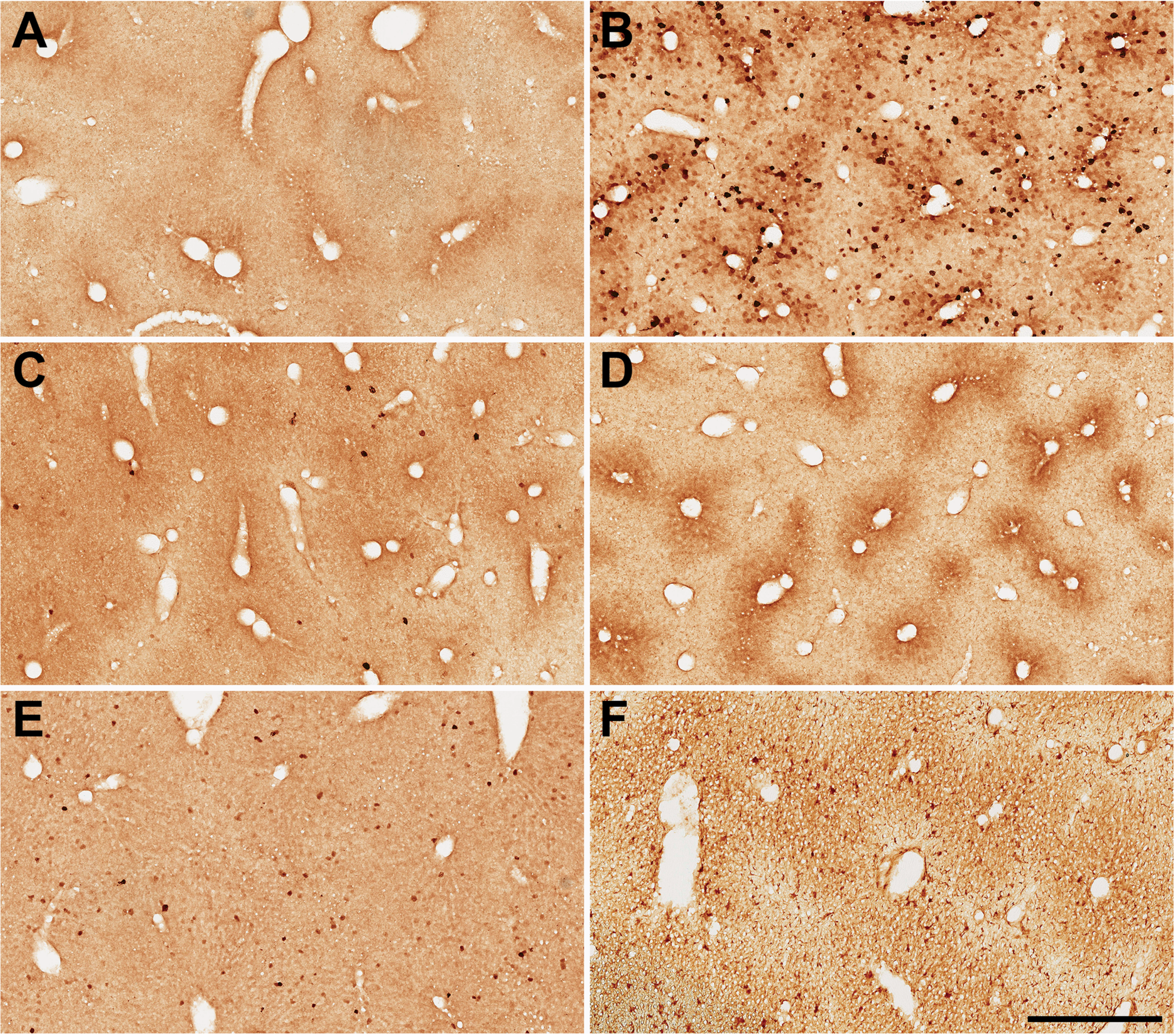

Suppl. Fig. 1 Transduction of cells in the liver following different delivery routes in mice, rats and NHPs. Different groups of 7 weeks-old C57BL6/J mice (3 females and 3 males) were left untreated (**A**) or received the AAV9P31-CMV-GFP vector by i.v. injection at $2x10^{13}$ vg/kg (**B**), or by i.c.v. injection at $1.2x10^{11}$ vg (**C**), or by unilateral intrastriatal injection at $6x10^{10}$ vg (**D**). Seven weeks-old rats (one female and one male) received the AAV9P31-CMV-GFP vector by iv injection at $2x10^{13}$ vg/kg (**E**). *Macaca fascicularis* (n= 2, male and female) were treated simultaneously with vectors AAV9P31-CMV-GFP ($2x10^{13}$ vg/kg), AAV9P31-CMV-tdT ($4x10^{13}$ vg) and AAV9P31-CMV-NL ($2x10^{11}$ vg) following i.v., i.c.v. and intraparenchymal injections (substantia nigra), respectively (**F**). One month later, animals were sacrificed and liver samples were processed for detection of GFP by immunohistochemistry. Scale bar is 500 µm in all panels.

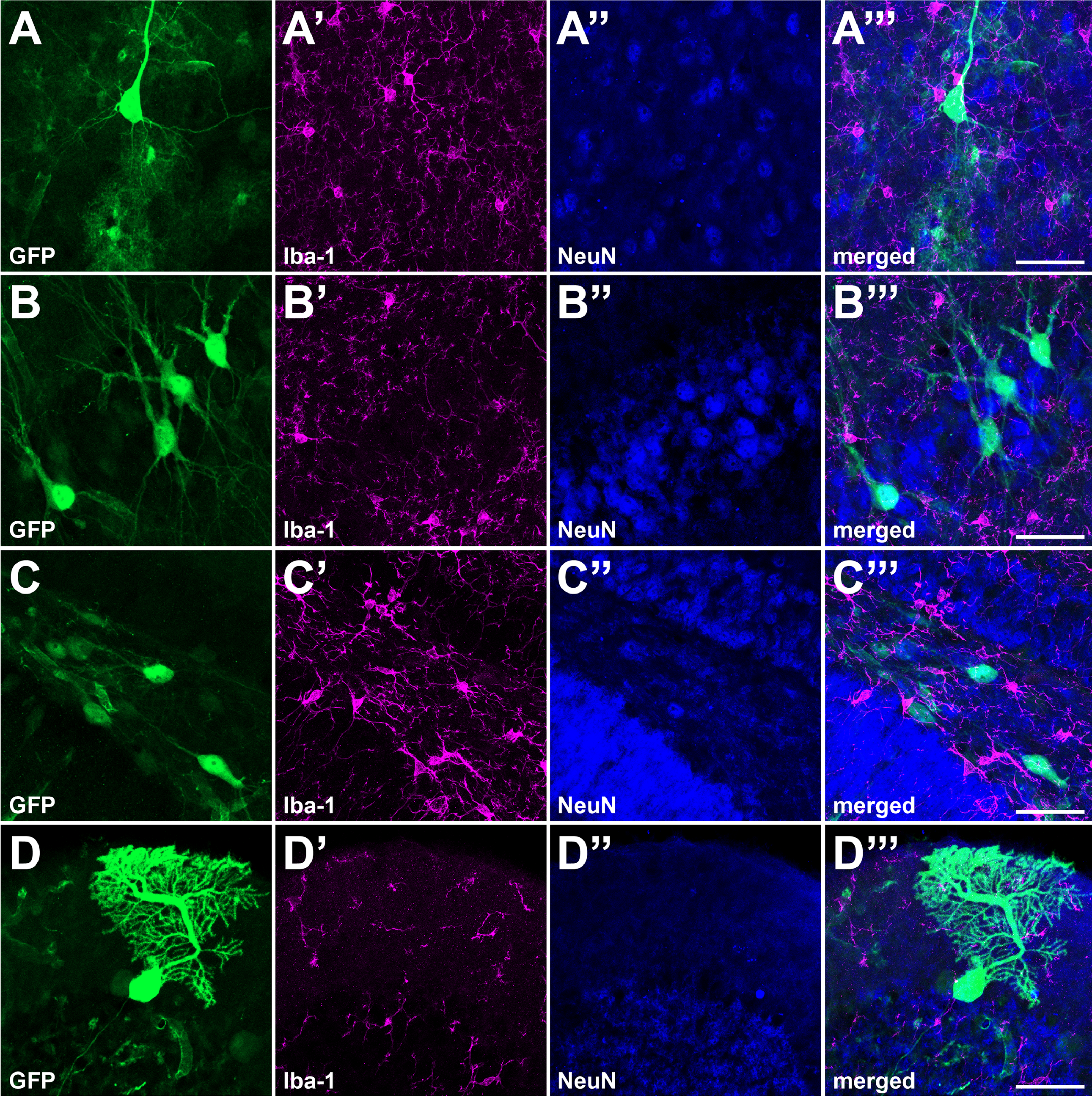

Suppl. Fig. 2 Lack of transduction of microglial cells upon i.v. delivery of AAV9P31-GFP in mouse brain cortex. C57 BL6/J mice (3 females and 3 males) received the AAV9P31-CMV-GFP vector by i.v. (retro-orbital) injection at $2x10^{13}$ vg/kg. One month later, mice were sacrificed and brain samples were processed for immunofluorescence using antibodies against GFP (green) and the indicated cell markers: NeuN for neurons (blue) and Iba1 for microglia (purple). GFP expression was never found in microglial cells in the cerebral cortex (**A-A’’’**), hippocampal formation (panels **B-B’’’** illustrating the CA1 field; **C-C’’’** taken from the dentate gyrus), and cerebellar cortex (D-D’’’). Staining with NeuN was used to define cortical layering (panels A’’, B’’, C’’ and D’’).

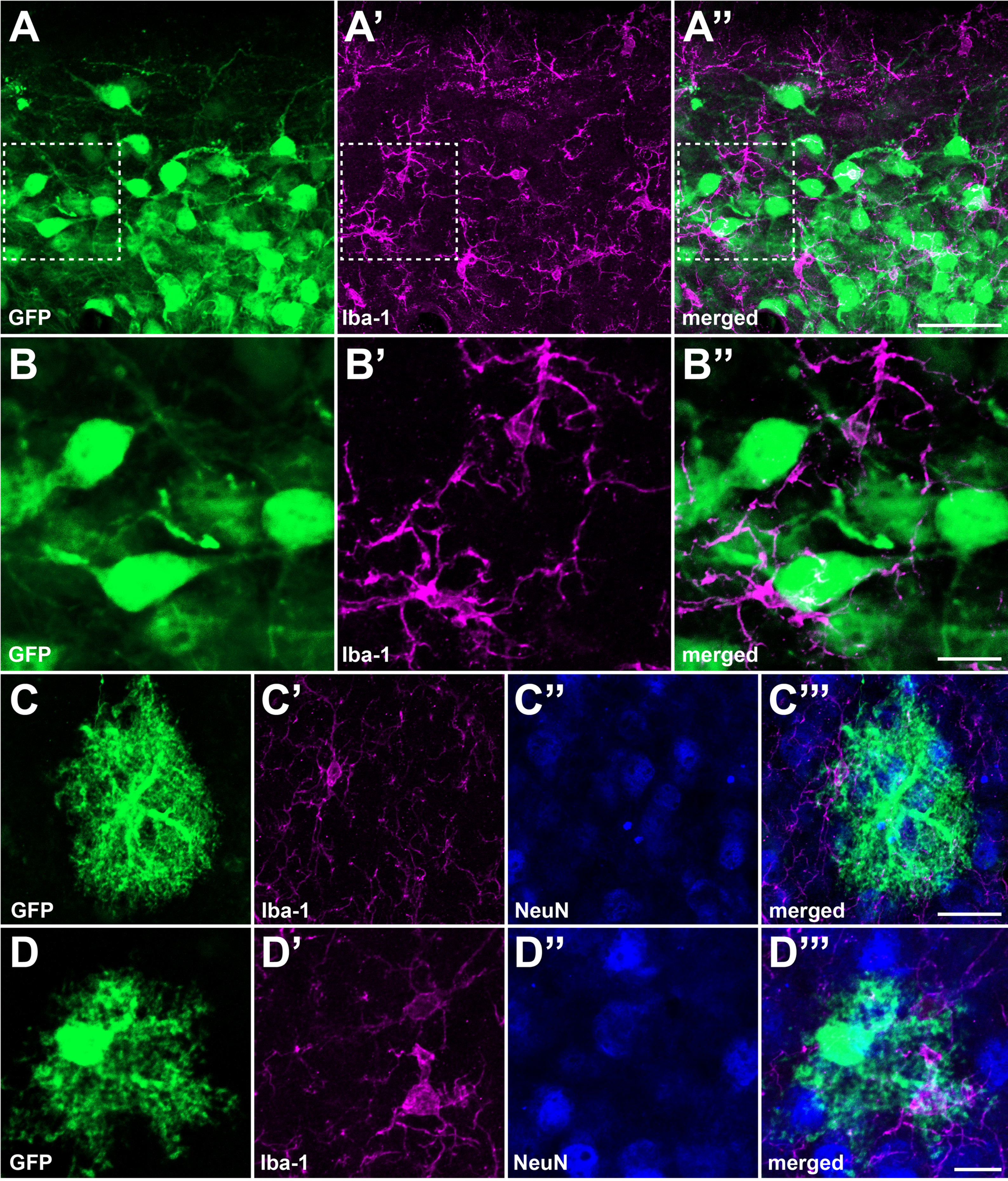

Suppl. Fig. 3 Lack of transduction of microglial cells upon i.v. delivery of AAV9P31-GFP in mouse subcortical regions. C57BL6/J mice (3 females and 3 males) received the AAV9P31-CMV-GFP vector by i.v. (retro-orbital) injection at $2x10^{13}$ vg/kg. One month later, mice were sacrificed and brain samples were processed for immunofluorescence using antibodies against GFP (green) and the indicated cell markers: NeuN for neurons (blue) and Iba1 for microglia (purple). GFP expression was never found in microglial cells in any subcortical brain area examined. Illustrative examples taken from the paraventricular nucleus of the hypothalamus are shown in panels **A-A" & B-B"** (insets). A similar lack of transduction of microglial cells was found in the cerebral cortex, where astrocytes were the most abundantly transduced cellular phenotype (panels **C-C"' & D-D"'**).

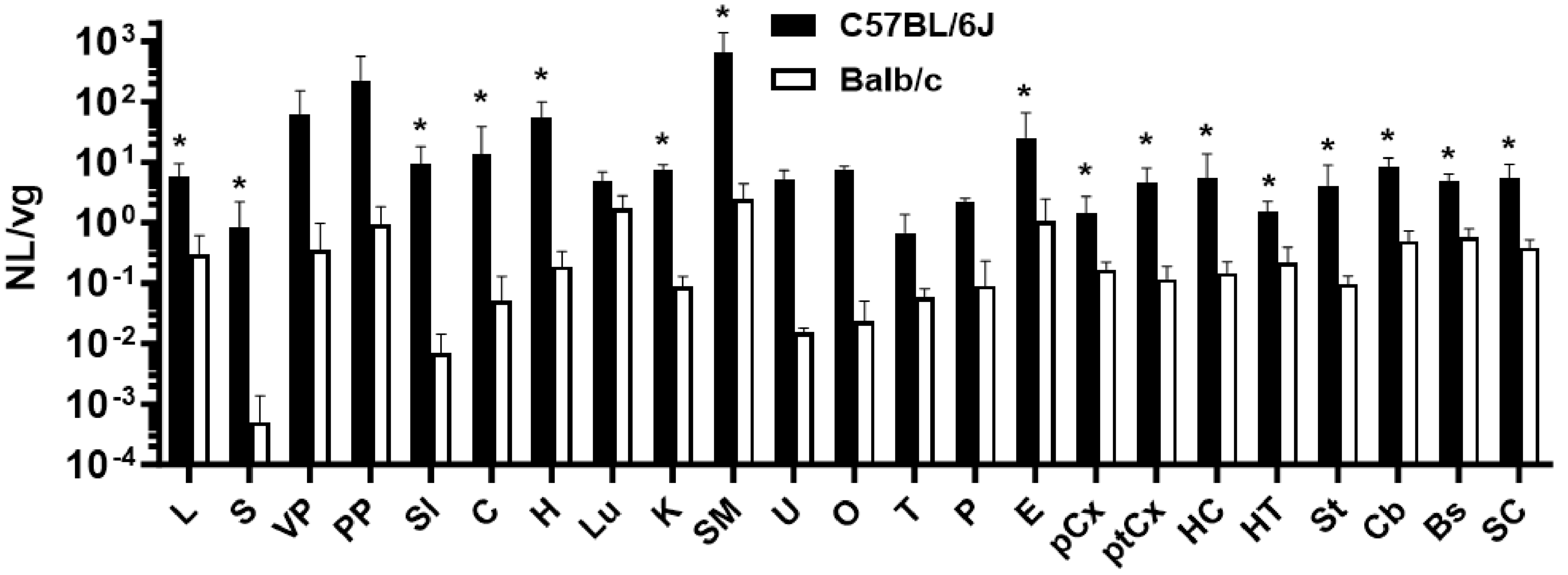

Suppl. Fig. 4 Ratio of NanoLuc activity and viral genomes in tissues of mice treated with i.v. injections of AAV9P31-CMV-NL. Seven weeks-old C57BL/6J and Balb/c mice (n= 6, male and female) received the AAV9P31-CMV-NL vector by i.v. (retro-orbital) injection at $2x10^{13}$ vg/Kg. One month later, mice were sacrificed for dissection of different CNS structures and peripheral organs. One portion of the tissue samples was used for quantification of luciferase activity ex vivo and other portion was processed DNA isolation and quantification of viral genomes by qPCR. The graph shows the NanoLuc activity (expressed as RLU/mg tissue) divided by the vector content (expressed in copies/µg DNA) in each tissue. *$p<0.05$, Mann Whitney U test.

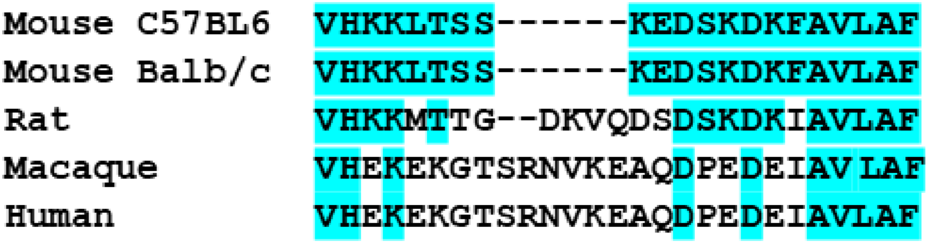

Suppl. Fig. 5 Protein sequence alignment of carbonic anhydrase IV (CA-IV) from different species. Blue color indicates aminoacids identical to the C57BL/6 CA-IV in the region described to bind to the AAV9P31 capsid (residues 141-161). Alignments generated by the Clustal Omega server.
